# An Activated Glassy Carbon Electrode for Rapid, Simple, and Sensitive Voltammetric Analysis of Diclofenac in Tablets

**DOI:** 10.3390/molecules30122530

**Published:** 2025-06-10

**Authors:** Katarzyna Tyszczuk-Rotko, Aleksy Keller, Aleksandra Liwak

**Affiliations:** Institute of Chemical Sciences, Faculty of Chemistry, Maria Curie-Skłodowska University in Lublin, 20-031 Lublin, Poland; alekskeller@op.pl (A.K.); liwakaleksandra@gmail.com (A.L.)

**Keywords:** diclofenac, stripping voltammetry, non-steroidal anti-inflammatory drug, an activated glassy carbon electrode, pharmaceutical preparations

## Abstract

This paper proposes an environmentally friendly sensor for determining trace amounts of diclofenac (DCF)—an activated glassy carbon electrode (aGCE). Such a sensor was achieved by simple surface activation of a glassy carbon electrode to perform five cyclic voltammetric scans from −1.5 to 2.5 V at a scan rate (υ) of 100 mV/s in 0.1 M NaOH. This type of activation results in the formation of surface functional groups, which provide several advantages such as the creation of new active sites, the improvement of electron transfer dynamics, and sensor electrocatalytic activity. The electrode prepared in this way was used to develop a new differential pulse adsorptive stripping voltammetric procedure (DPAdSV) for rapid, simple, and sensitive DCF analysis. Thanks to this procedure, the following calibration curve range was obtained: 1–100 nM with low detection and quantification limits of 0.25 and 0.83 nM, respectively. To show the practical application of the method, DCF was successfully determined in commercially available pharmaceutical preparations with the standard addition method.

## 1. Introduction

Diclofenac (DCF, 2-[2-(2,6-dichloroanilino)phenyl]acetic acid) is classified as a non-steroidal anti-inflammatory drug (NSAID) and is commercially sold in sodium salt form. Pharmacies offer a wide range of pharmaceuticals containing DCF (or its salts) as an active substance, which are sold in both tablet and gel form. It has been introduced into treatment since 1979 and is currently one of the most commonly prescribed pain killers in the world, along with ibuprofen, acetylsalicylic acid, or naproxen. Its basic role in the human body (like other drugs from the NSAID group) is to inhibit the oxygenase enzyme, which is responsible for the synthesis of prostaglandins from cell membrane lipids. Due to this mechanism of action, diclofenac has anti-inflammatory, analgesic, antipyretic, and platelet aggregation-inhibiting effects. Unlike other NSAIDs, diclofenac also inhibits lipoxygenase activity, which is responsible for the formation of other pro-inflammatory factors, the so-called leukotrienes. In addition, diclofenac is a drug that acts more strongly than the equally commonly used aspirin. This drug is also used synergistically with nucleoside metabolism inhibitors to treat certain types of cancer and inhibit tumor growth. After oral ingestion, DCF is rapidly absorbed and its half-life is estimated to be from one to two hours [1,2,3]. The literature states that DCF has toxic effects and it affects aquatic and terrestrial ecosystems in such a way that it affects bacteria, invertebrates, and algae and causes physiological changes in animals. It is worth mentioning that for human health, DCF can cause side effects such as nausea, difficulty breathing, allergic reactions, eye swelling, rapid heartbeat, etc. [1,2,3]. Its popularity and frequent human consumption/use for pain relief has resulted in its presence in sewage. Moreover, due to the creation of new pharmaceutical preparations with different diclofenac contents, there is a need to develop a quick and simple method for determining this compound.

Many methods have been proposed in the literature for the determination of DCF, such as chromatographic methods: high performance liquid chromatography [4,5,6], gas chromatography [6], and high-performance thin layer chromatography [7,8]; spectroscopic: FT-Raman spectroscopy [9], spectrofluorimetry [10], ATR-FTIR [11], and UV spectrophotometry [12,13]; and electrochemical methods such as potentiometry [14] and voltammetry [1,2,15,16,17,18,19,20,21,22,23,24,25,26,27]. In most cases, voltammetric methods are used, which use different types of sensors. These methods, in comparison to chromatographic and spectroscopic methods, do not use expensive and difficult-to-use apparatus. In addition, in most cases these methods do not require the use of complex and toxic chemical reagents, as in chromatography, and there is no need to use various types of complicated stationary phases. One significant advantage of voltammetric methods is the possibility of miniaturization, which consequently allows for analysis to be carried out in field conditions. These methods are known for achieving the lowest detection (LOD) and quantification limits (LOQ) by using stripping methods such as anodic (ASV), cathodic (CSV), and adsorptive (AdSV) stripping voltammetry—the last one is mainly used for the determination of organic compounds [28,29].

In the literature, many developed voltammetric methods can be found in which different types of electrodes have been used, and we compare these methods in Table 1 [1,2,15,16,17,18,19,20,21,22,23,24,25,26,27]. It can be seen that the obtained ranges of calibration curves and the detection and quantification limits vary from nanomolar to millimolar values. Recently, there has been great interest in preparing modified electrodes and testing and checking them in trace analysis. Modification can be achieved by dropping different inks, such as multiwalled carbon nanotubes [1,2,15,16,18,20,27] and, as can be seen, this type of modification predominates in the vast majority of cases of DCF voltammetric determination. The best results are obtained with MWCNT modification, but the preparation of electrodes in this way is very time-consuming (as compared to other types of modification). In addition, this introduces even more chemical reagents for analysis, and they are not always environmentally friendly.

In 2020, Abdel-Aziz et al. first used a new type of modification—an activation—and their team successfully used an activated glassy carbon electrode to determine 4-nitrophenol and dopamine in real samples [30,31]. Activation was achieved by performing five cyclic voltammetry scans in 0.1 phosphate-buffered saline solution (PBS). Abdel-Aziz et al. discovered that such activation on carbon surfaces can create oxygen-containing surface functional groups (O-SFGs) such as carboxylic groups or phenolic groups. Electrodes prepared in this way provide several advantages, such as better electron transfer kinetics or lower charge transfer resistance compared to bare carbon electrodes. Inspired by their work, our team performed voltammetric trace analysis of compounds such as methyl jasmonate [32] and simultaneous determination of lead and cadmium [33] with activation in different solutions such as sodium hydroxide or sulfuric acid. Our research shows that the carbon surface of the electrode during activation additionally gains polarity, which influences the interactions between the analyte and other deposited compounds.

Our main goal was to achieve rapid and simple voltammetric analysis, which is characterized by simple and fast preparation of the electrode, the lowest possible usage of chemical reagents, and the use of electrodes/reagents that are environmentally friendly. It is worth mentioning that activated electrodes have already been used in trace analysis of DCF [25], but they did not provide such good results as those obtained in our work. This is due, among other things, to the use of a different procedure and electrolyte for electrochemical activation. Furthermore, after optimizing the sensor preparation procedure and DCF determination, it was used for analysis in natural samples.

## 2. Results and Discussion

### 2.1. Activation of the Electrode Surface

**Figure 1:**
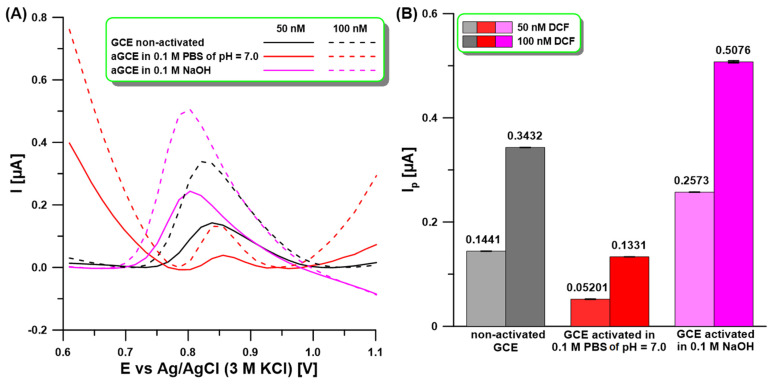
(**A**) DPAdSV measurements for 50 and 100 nM DCF at the non-activated GCE and activated in 0.1 M PBS of pH = 7 or NaOH by five CV scans from −1.5 to 2.5 V at ν of 100 mV/s. (**B**) The anode peak current (I_p_) of DCF obtained using the GCE and aGCE with SD values (n = 3). DPAdSV parameters: E_acc._ of −0.25 V, t_acc._ of 60 s, ΔE_A_ of 125 mV, υ of 175 mV/s, and t_m_ of 10 ms.

Our previous studies have shown that electrochemical activation of the GCE surface in 0.1 M NaOH or 0.1 phosphate-buffered saline solution (PBS) of pH = 7 by five cyclic voltammetric (CV) scans from −1.5 to 2.5 V at a scan rate (ν) of 100 mV/s contributes to the enhancement of the analytical signal of the determined methyl jasmonate or Cd(II) and Pb(II) ions [32,33]. This is related to the fact that the functionalization of the GCE surface by oxygen-containing groups not only creates new active sites but also improves electron transfer dynamics and electrocatalytic activity. The formation of these groups has been well described in other papers [30,31]. In the literature, there have been attempts to use electro-activated carbon electrodes for DCF determination, but an activation procedure at a constant potential of 2.0 V for 60 s has been used [25]. In preliminary studies, the differential pulse adsorptive stripping voltammetric (DPAdSV) analytical signals of DCF (50, 100 nM) at a non-activated GCE and the GCE activated according to procedures applied in our previous articles [32,33] (Figure 1A,B) were compared. The DCF was accumulated at a potential of −0.25 V for 60 s. As can be seen, clearly visible and shaped DCF peaks were obtained on all tested electrodes. However, only when activation in 0.1 M NaOH was used was a significant increase in DCF analytical signals obtained. Therefore, the aGCE obtained in this solution was used in further studies. It is worth adding that CV, electrochemical impedance spectroscopy (EIS), and X-ray photoelectron spectroscopy (XPS) studies of the GCE and the aGCE activated in 0.1 M NaOH were conducted in our previous paper [32]. These studies confirmed that activation contributes to the formation of hydroxyl, carbonyl, and carboxyl functional groups. Their combined influence contributes to enhancement in the electrocatalytic activity of the aGCE.

### 2.2. Voltammetric Behavior of DCF

**Figure 2:**
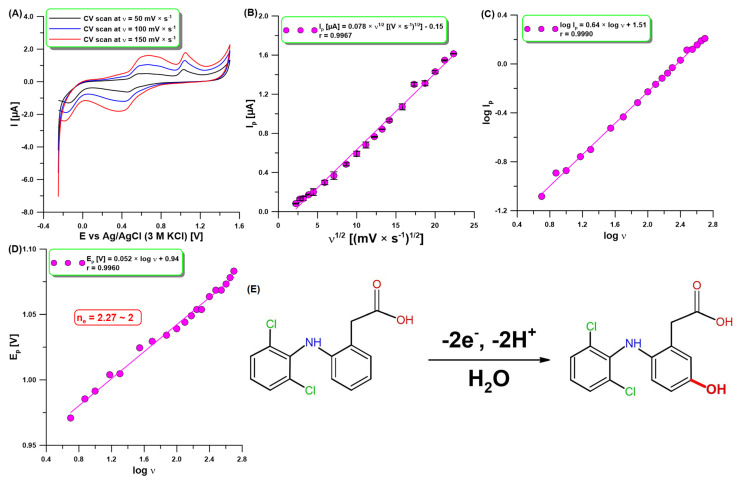
(**A**) CV results at the aGCE in 0.1 M acetate buffer of pH = 4 and 10 µM DCF. The relationship between (**B**) I_p_ and υ^1/2^, (**C**) log I_p_ and log υ, and (**D**) E_p_ and log υ (υ: 5–500 mV/s). (**E**) The proposed oxidation mechanism of DCF at the aGCE. The SD values were calculated for n = 3.

Figure 2A shows the CV results for the analysis of DCF (10 µM) at the aGCE with selected three υ of 50, 100, and 150 mV/s. As can be seen, there are two anodic peaks at 0.60 and 1.04 V (υ of 100 mV/s). The first one formed a reversible couple with a cathodic peak at 0.42 V (υ of 100 mV/s) due to the oxidation product of DCF, which is electrochemically active [25,34,35]. The reaction products did not block the electrode surface because the electrode was cleaned before each measurement during recording of the voltammogram to a high positive potential (1.5 V). The second anodic peak confirmed that DCF is irreversibly oxidized, giving rise to an oxidation peak when the sweep was initiated in the positive direction. The DCF oxidation peak current (I_p_) was found to increase with increasing sweep rates (υ: 5–500 mV/s). The relationship between I_p_ and υ^1/2^ was directly proportional (r = 0.9967), which demonstrates that the oxidation reaction of DCF at the aGCE was a diffusion-controlled process (Figure 2B). However, the slope value of 0.64 in the relationship between log I_p_ and log υ (Figure 2C) is higher than the theoretical one of 0.5. This result shows that the nature of the DCF oxidation process at the aGCE is adsorption–diffusion controlled [36]. Moreover, the number of electrons involved in the DCF oxidation process was calculated from the slope of the peak potential (E_p_) vs. log υ plot (Figure 2D) using Laviron’s equation [37]. The value determined is equal to 2.27, which proves that two electrons are involved in this process. These results are consistent with the literature data [27,35]. The DCF is most probably oxidized to 5-hydroxydiclofenac by loss of 2e^−^ and 2H^+^ (Figure 2E) [27,35].

**Figure 3:**
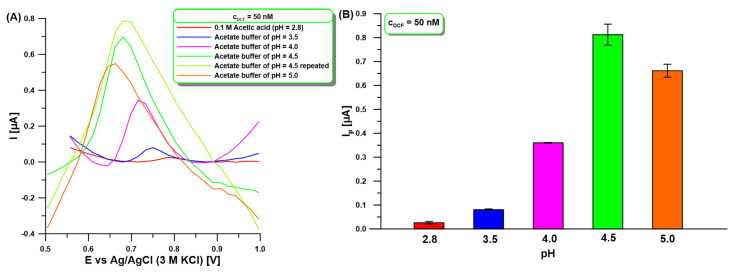
(**A**) DPAdSV measurements registered in supporting electrolyte (0.1 M acetic acid and acetate buffer) with different pH values of 2.8 to 5.0 in the presence of 50 nM DCF. (**B**) The relationship between I_p_ and pH of supporting electrolyte. DPAdSV parameters: E_acc._ of −0.25 V, t_acc._ of 60 s, ΔE_A_ of 125 mV, υ of 175 mV/s, and t_m_ of 10 ms. The SD values were calculated for n = 3.

Furthermore, the effect of the pH of the supporting electrolyte (0.1 M acetic acid and acetate buffer) on the 50 nM DCF signal at the aGCE with different pH values of 2.8 to 5.0 was studied (Figure 3). The results indicate that DCF oxidation peak current (I_p_) increases with increasing pH from 2.8 to 4.5 and then the peak current decreases (Figure 3A). The diclofenac molecule assumes protonated and deprotonated forms around pH = 4 (pKa = 4.15) [38]. The decrease in peak current at pH values lower than pK_a_ is attributed to the protonation of DCF. At pH near pK_a_, the neutral form of DCF predominates, leading to an increase in peak current. However, at pH values above pKa, the peak current decreases due to DCF deprotonation. Moreover, the DCF peak potential is shifted positively along with the decrease in pH (Figure 3A), indicating a higher oxidation over-potential occurred at lower pH values [39]. For further measurements 0.1 M acetate buffer with pH = 4 was selected, although the DCF current intensity was higher for pH = 4.5 (Figure 3B). This choice was dictated by the change in peak shape from measurement to measurement at pH above 4 (Figure 3A). This contributed to the difficulty of measuring analytical signals.

### 2.3. Step of DCF Accumulation

**Figure 4:**
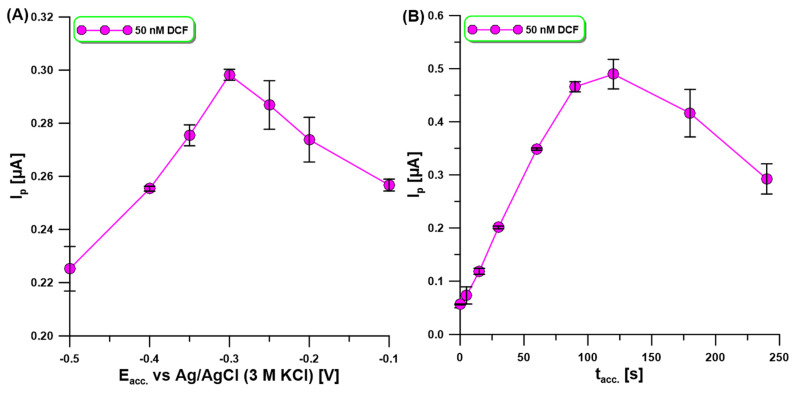
The effect of (**A**) E_acc_ and (**B**) t_acc_ on the oxidation peak current of 50 nM DCF. DPAdSV parameters: t_acc._ of 60 s (**A**), E_acc_ of −0.3 V (**B**), ΔE_A_ of 125 mV, υ of 175 mV/s, and t_m_ of 10 ms. The SD values were calculated for n = 3.

DPAdSV measurements in 0.1 M acetate buffer of pH = 4 at the aGCE were carried out to characterize the effect of analyte accumulation potential (E_acc_) and time (t_acc_) on the oxidation peak current of 50 nM DCF. In the first stage of the experiment, E_acc_ was changed from −0.1 to −0.5 V, but t_acc_ was constant and equal to 60 s. As shown in Figure 4A, the highest oxidation peak current of DCF with satisfactory repeatability was attained at a potential of −0.3 V. Then, t_acc_ was changed from 0 to 240 s (Figure 4B). The highest DCF signal was obtained for t_acc_ of 120 s, while above this time the signal decreased. Most probably due to the lack of active sites at the trough, DCF could accumulate. Nevertheless, t_acc_ equal to 90 s was chosen for the study, due to better signal repeatability and a slight difference in its height.

### 2.4. Signal Registration Technique Parameters

**Figure 5:**
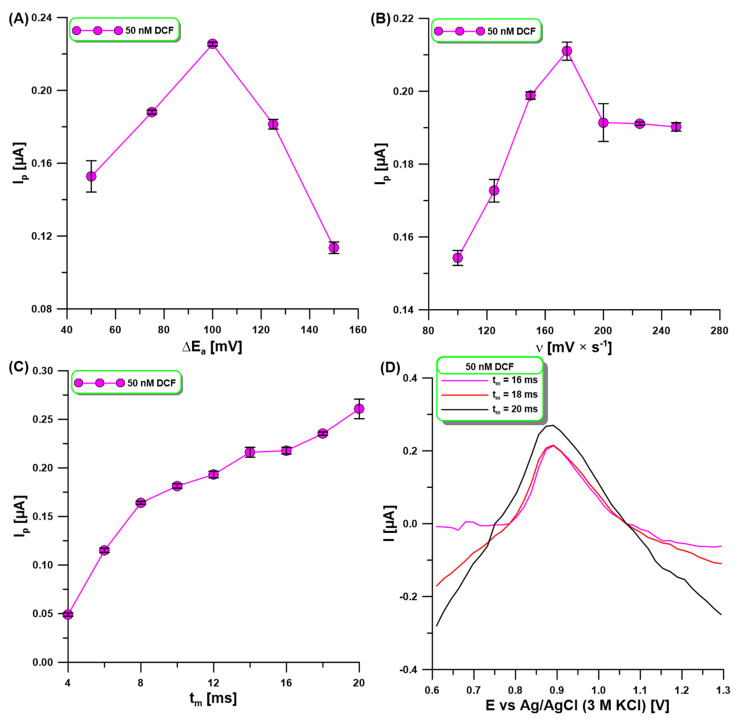
The effect of ΔE_A_ (**A**), υ (**B**), and t_m_ (**C**) on the oxidation peak current of 50 nM DCF. DPAdSV measurements registered at three different t_m_ (**D**). DPAdSV parameters: E_acc._ of −0.3 V and t_acc._ of 90 s. The SD values were calculated for n = 3.

The signal registration technique (DPV) parameters, the amplitude (ΔE_A_), the scan rate (υ), and the modulation time (t_m_)) were optimized in order to achieve optimal sensitivity. The influence of ΔE_A_ (50–150 mV) on the oxidation peak current of 50 nM DCF was evaluated (Figure 5A). The DCF signal was observed to increase with increasing ΔE_A_ up to 100 mV, while later the signal decreased. For the selected value of ΔE_A_ of 100 mV, ʋ was now varied from 100 to 250 mV/s, and the changes in the 50 nM of DCF peak were observed (Figure 5B). With the increase in υ, the DCF signals increased, reaching the highest intensity at ʋ of 175 mV/s. The effect of t_m_ on the oxidation peak current of 50 nM DCF was evaluated in the range of 4–20 ms (Figure 5C). A continuous increase in the DCF signal was observed with the increase in t_m_ in the studied range. However, the increase in t_m_ also contributed to the deterioration in the peak shape and the difficulty in its measurement (Figure 5D). For this reason, a t_m_ of 16 ms was selected.

### 2.5. Selectivity, Repeatability, and Reproducibility

**Figure 6:**
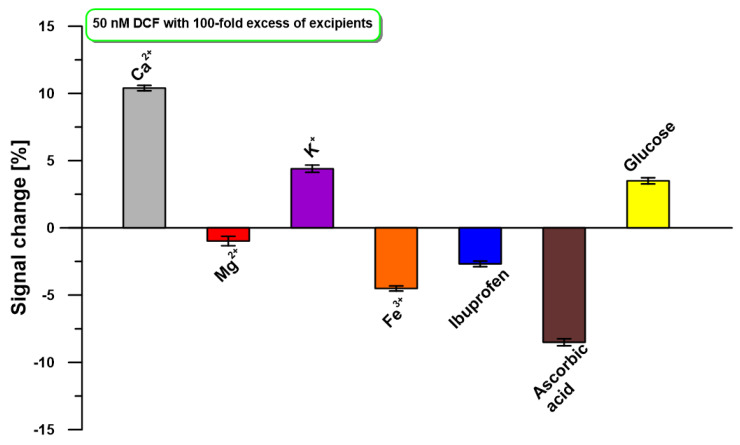
The influence of a 100-fold excess of selected interferents on the oxidation peak current of 50 nM DCF. DPAdSV parameters: E_acc._ of −0.3 V, t_acc._ of 90 s, ΔE_A_ of 100 mV, υ of 175 mV/s, and t_m_ of 16 ms.

The developed DPAdSV procedure at the aGCE was tested against common substances and ions presented in the tablets. The 100-fold excesses of Ca^2+^, Mg^2+^, K^+^, Fe^3+^, ibuprofen, ascorbic acid, and glucose in relation to the DCF concentration (50 nM) were studied. As illustrated in Figure 6, no significant current variation (±10% signal change) was observed.

Repeatability was evaluated with successive 50 nM DCF measurements (n = 10) at the aGCE. The obtained RSD value of 4.7% confirms satisfactory signal repeatability. Moreover, electrode-to-electrode reproducibility was calculated for three independently prepared aGCEs based on the oxidation peak current of 50 nM DCF (n = 15). The RSD value of 7.9% confirms satisfactory reproducibility.

### 2.6. Analysis of Performance and Practical Application

**Figure 7:**
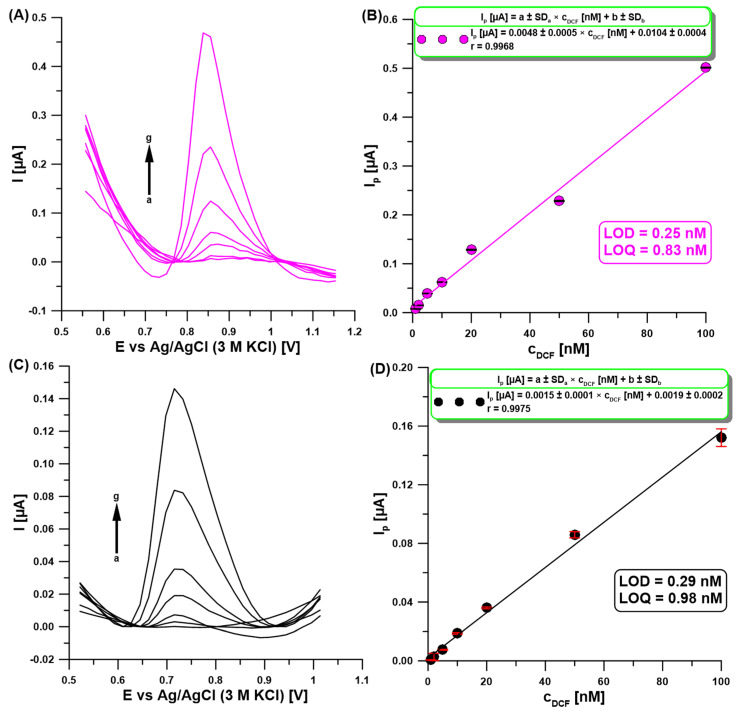
DPAdSV measurements at the aGCE (**A**) and a bare GCE (**C**) in 0.1 M acetate buffer with pH of 4 containing DCF (a → g, 1–100 nM). The calibration curve at the aGCE (**B**) and a bare GCE (**D**). DPAdSV parameters: E_acc._ of −0.3 V, t_acc._ of 90 s, ΔE_A_ of 100 mV, υ of 175 mV/s, and t_m_ of 16 ms. The SD values were calculated for n = 3.

To validate the quantitative determination capability of the developed DPAdSV procedure with the aGCE, a gradient concentration of DCF under optimized conditions was analyzed (Figure 7A). The obtained calibration curve is characterized by a wide linear range from 1 to 100 nM of DCF (Figure 7B). The equations LOD = 3 SD_a_/b and LOQ = 10 SD_a_/b (SD_a_—standard deviation of intercept (n = 3), b—slope of the linear regression equation) were used to determine the limits of detection (LOD = 0.25 nM) and quantification (LOQ = 0.83 nM) of DCF. Table 1 compares the voltammetric procedures for the DCF analysis in various samples [1,2,15,16,17,18,19,20,21,22,23,24,25,26,27]. As can be seen, the developed method for DCF determination at the aGCE does not require a complicated sensor preparation procedure, which contributes to reducing the consumption of reagents. Moreover, it offers a wide linear range of the calibration curve and one of the lowest LODs.

Moreover, the performance of a series of measurements at a bare GCE under optimized conditions for the aGCE (Figure 7C) showed that electrode activation improved the sensitivity of DCF determination (0.0015 vs. 0.0048 µA/nM, respectively) and slightly improved detection and quantification limits (Figure 7D).

The developed procedure at the aGCE was applied for DCF determination in commercially available tablets containing 25 mg/tablet DCF (tablets A and B). The results (Table 2) show a satisfactory degree of precision (coefficients of variation: 3.2 and 5.7%) and accuracy (recoveries: 97.6 and 102%) for the developed DPAdSV procedure.

## 3. Materials and Methods

### 3.1. Instrumentation

An electrochemical analyzer (µAutolab, Utrecht, Netherlands, Eco Chemie) was applied for the voltammetric experiments. The glassy carbon electrode (GCE, geometric area of 3.14 mm^2^, Mineral, Warsaw, Poland) was polished using 0.3 µm alumina slurry on a Buehler polishing pad (Lake Bluff, IL, USA). A silver/silver chloride (3 M KCl) electrode and a Pt wire were used as the reference and auxiliary electrodes, respectively.

### 3.2. Reagents

2-[(2,6-dichlorophenyl)amino] benzeneacetic acid sodium salt (DCF) was purchased from the Sigma-Aldrich company (Saint Louis, MO, USA) and was dissolved in deionized water to prepare a 0.001 M stock solution. This solution was diluted as required for individual experiments using deionized water and stored at 4 °C in the dark until used. The supporting electrolyte solution was 0.1 M acetate buffer of pH = 4 prepared from Merck (Darmstadt, Germany) reagents. Interferences were tested using standard solutions of Ca^2+^, Mg^2+^, K^+^, Fe^3+^, ibuprofen, ascorbic acid, and glucose (Merck, Darmstadt, Germany).

The pharmaceuticals (tablets A and B) were prepared by the following procedure. Three tablets A or B were weighed, and then the average mass per tablet was determined. The tablets were carefully ground to a fine powder and then a quantity of homogeneous powder equivalent to the average mass per tablet was dissolved in 50 mL of 0.1 M NaOH by sonication for 2 h. Next, the appropriate amount of such prepared sample (0.1 µL) was added to the supporting electrolyte in the voltammetric cell.

### 3.3. aGCE Preparation and DCF Analysis

An electrochemically activated glassy carbon electrode (aGCE) was created by simple surface activation of the GCE surface by performing five cyclic voltammetric scans from −1.5 to 2.5 V at a scan rate (υ) of 100 mV/s in 0.1 M NaOH [32]. Then, DPAdSV measurements of DCF were performed in 0.1 M acetate buffer of pH = 4. DCF was accumulated at the aGCE surface at a potential of −0.3 V (E_acc._) for 90 s (t_acc._) under stirring. DPAdSV signals were registered with an amplitude (ΔE_A_) of 100 mV, a scan rate (υ) of 175 mV/s, and a modulation time (t_m_) of 16 ms. The background was subtracted from each measurement.

## 4. Conclusions

In this paper, an environmentally friendly sensor for simple and rapid determination of trace amounts of diclofenac (DCF)—an activated glassy carbon electrode (aGCE)—is proposed. It has been found that the electrochemical activation of the GCE surface in 0.1 M NaOH by performing five cyclic voltammetric scans from −1.5 to 2.5 V at a scan rate (υ) of 100 mV/s contributes to the acquisition of the highest differential pulse adsorptive stripping voltammetric (DPAdSV) signals of DCF. It is connected with the formation of oxygen-containing groups on the GCE surface, which provides several advantages such as the creation of new active sites and the improvement of electron transfer dynamics and sensor electrocatalytic activity. The developed method does not require a complicated sensor preparation procedure, which contributes to reducing the consumption of reagents. Moreover, it offers a wide linear range in calibration curve values and some of the lowest LODs and LOQs. The analytical performances of the aGCE were satisfactory, as evidenced by the repeatability, reproducibility, and selectivity of the procedure. The practical applicability was positively confirmed with commercially evaluable tablets.

## Figures and Tables

**Table 1:** Comparison of voltammetric procedures for DCF analysis in various samples.

Electrode	Technique	Linear Range [nM]	LOD [nM]	Application	Reference
GCE/MWCNT/AuNP	SWAdSV	30–200,000	20.0	Tablet and human urine	[1]
CPE/MWCNT/poly(Gly)	DPV	500–1000	79.6	Tablet and urine	[2]
GRP/MWCNT/CuNP@OA	DPV	17,410–206,450	57.7	Drinking water and milk	[15]
CPE/VF/MWCNT	SWV	500–600,000	200	Tablet and urine	[16]
CNTPE/IL	SWV	30–750,000	90	Tablet and urine	[17]
GCE/MWCNT/Cu(OH)_2_	DPV	180–119,000	40.0	Tablet, human and fish blood serum, and seawater	[18]
CPE/chitosan/TiO_2_NP	DPAdSV	20–10,000 10,000–100,000	13.0	Tablet and synthetic urine	[19]
ITO/IL/Asp-MWCNT	CV	40,000–160,000	9.40	Water samples	[20]
Mo@GO/CS-V_2_O_5_	SWV	10–200	10.2	Human plasma and urine	[21]
GCE/poly(Co(Phen)_2_)	SWAdSV	1000–250,000	56.0	Pharmaceutical, milk, serum, and urine	[22]
CPE/Ru-TiO_2_	SWV	100–2000	1.48	Urine sample	[23]
SPCPE/GE/Gd_2_O_3_	DPV	5890–66,700	28.0	-	[24]
aGCE *	DPV	10–50	5.03	-	[25]
aSPCE	DPV	67–490	22.0	-	[25]
CPE/M-ChsNC	DPV	25–4000	7.00	Tablet and human serum	[26]
SPCE/MWCNTs-COOH	DPAdSV	0.1–10	0.028	River water	[27]
aGCE	DPAdSV	1–100	0.25	Tablet	This work

GCE/MWCNT/AuNP—glassy carbon electrode modified with multiwalled carbon nanotubes and gold nanoparticles; GRP/MWCNT/CuNPs@OA—graphene electrode modified with multi-walled carbon nanotube and copper nanoparticles blended with oleic acid; CPE/VF/MWCNT—carbon paste electrode modified with vinylferrocene and multiwalled carbon nanotubes; CNTPE/IL—carbon nanotube paste electrode modified with ionic liquid; GCE/MWCNT/Cu(OH)_2_—glassy carbon electrode modified with multiwalled carbon nanotubes and copper hydroxide; CPE/chitosan/TiO_2_NP—carbon paste electrode coated with chitosan and modified with TiO_2_ nanoparticles; ITO/IL/Asp-MWCNT—indium tin oxide electrode loaded with ionic liquid and asparagine-functionalized multi-walled carbon nanotubes; Mo@GO/CS-V_2_O_5_—molybdenum-doped graphene oxide nanorod-anchored carbon spheres/vanadium pentoxide nanocomposites; GCE/poly(Co(Phen)_2_)—glassy carbon electrode modified with poly [cobalt(II)bis-(1,10)-phenanthroline]; CPE/Ru-TiO_2_—carbon paste electrode modified with ruthenium-doped titanium dioxide; SPCPE/GE/Gd_2_O_3_—screen-printed carbon paste electrode modified with graphene and gadolinium oxide composite; CPE/M-ChsNC—carbon paste electrode modified with magnetic chitosan nanocomposite; SPCE/MWCNTs-COOH—screen-printed carbon electrode modified with carboxyl functionalized multiwalled carbon nanotubes; aGCE—activated glassy carbon electrode; aSPCE—activated screen-printed carbon electrode; SWAdSV—square-wave adsorptive stripping voltammetry; DPV—differential pulse voltammetry; DPAdSV—differential pulse adsorptive stripping voltammetry; CV—cyclic voltammetry; *—the use of a different procedure and electrolyte for electrochemical activation than in this work.

**Table 2:** The results of DCF determination in tablets.

DCF Content [mg/tablet] ± SD (n = 3)			
Sample (Content)	Found DPAdSV	Coefficient of Variation * [%]	Recovery ** [%]
A (25)	24.4 ± 0.77	3.2	97.6
B (25)	25.5 ± 1.45	5.7	102

* Coefficient of variation [%] = (SD × 100)/Found DPAdSV; ** Recovery [%] = (Found DPAdSV × 100)/Content specified by the manufacturer.

## Data Availability

The original contributions presented in this study are included in the article. Further inquiries can be directed to the corresponding author.
